# Unravelling the Role of Socioeconomic Forces in the Early Stage of COVID-19 Pandemic: A Global Analysis

**DOI:** 10.3390/ijerph18126340

**Published:** 2021-06-11

**Authors:** Kostas Rontos, Maria-Eleni Syrmali, Luca Salvati

**Affiliations:** 1 Department of Sociology, University of the Aegean and Greece, University Hill, 81100 Lesvos Mytilene, Greece; k.rontos@soc.aegean.gr (K.R.); mesyrmali@soc.aegean.gr (M.-E.S.); 2 Department of Economics and Law, University of Macerata, Corso Cefalonia, 70, AP 63023 Fermo, Italy

**Keywords:** COVID-19 pandemic, healthcare, health policy, indicators, contagions

## Abstract

The COVID-19 pandemic has rapidly evolved into an acute health crisis with extensive socioeconomic and demographic consequences. The severity of the COVID-19 pandemic requires a refined (and more comprehensive) understanding of virus dissemination over space, transmission mechanisms, clinical features, and risk factors. In line with this assumption, the present study illustrates a comparative, empirical analysis of the role of socioeconomic and demographic dimensions in the early stages of the COVID-19 pandemic grounded on a large set of indicators comparing the background context across a global sample of countries. Results indicate that—in addition to epidemiological factors—basic socioeconomic forces significantly shaped contagions as well as hospitalization and death rates across countries. As a response to the global crisis driven by the COVID-19 pandemic, all-embracing access to healthcare services should be strengthened along with the development of sustainable health systems supported by appropriate resources and skills. The empirical findings of this study have direct implications for the coordination of on-going, global efforts aimed at containing COVID-19 (and other, future) pandemics.

## 1. Introduction

The COVID-19 pandemic has rapidly evolved into the most acute health crisis and the greatest challenge humanity has encountered since World War ΙΙ [1]. As COVID-19 reached a pandemic level, placing significant strains on healthcare systems worldwide, most countries implemented stringent containment measures. In such conditions, social protection mechanisms became critical to help preserve individuals and communities from pandemic impacts [2].

Early detection of COVID-19 and prevention of onward transmission were assumed as crucial factors in containing the risk of importation from hotspot regions with a surplus of active cases. Governments were compelled to undertake preventive actions mitigating the risk of contracting the virus—including infections from asymptotic COVID-19 patients. In order to reduce virus dissemination, stringent quarantine, mandatory lockdown (either partial or total), and public health restrictions have been imposed by many governments at the global level [3]. Moreover, virus *transmissibility* was lowered through social distancing measures, travel bans, the closure of schools and universities, as well as cancellation of public events involving large-scale gatherings, such as sport events, or discotheques [4].

Despite significant alleviation and containment measures, COVID-19 had (and still has) a devastating impact on health systems, societies (from those of affluent economies to those in emerging or developing countries, with no exceptions), and individuals around the world. It was largely assumed that this pandemic has the potential to evolve into a severe (and global) socioeconomic and political crisis [5], requiring a prompt (short-term) response addressing and advancing both efficacy and representativeness of countries’ health sectors and additional, refined (medium-term) policies averting the outburst of a global human crisis more effectively [6]. However, the specific influence of basic socioeconomic forces on COVID-19 expansion and health impacts at the global scale is still under investigation. Considering the experience and knowledge accumulated in earlier studies addressing other infectious diseases [7], various analysis frameworks can be adopted to fulfil these objectives, from purely qualitative surveys to analyses based on more sophisticated quantitative methodologies. The intrinsic lack in recent literature linking macroscale factors of viruses’ (large scale) spread, may be explained with the assumption that sociodemographic measures and indicators were (and still are) occasionally regarded as variables of clinical interest in epidemiological research [8]. This assumption reflects a significant limit of investigations on the evolution of infectious diseases in diversified socioeconomic contexts [9].

Based on these premises, our study contributes to the epidemiological literature on the COVID-19 pandemic, by focusing on the impact diversified contextual variables (namely socioeconomic, demographic, geographic, and climatic) have on (i) the intrinsic spread of the virus (assessing the total number of infected people, i.e., contagions), (ii) the pressure on health systems (considering the number of hospitalized patients), and (iii) the societies themselves (according with the total number of people infected by the virus who finally died during the first pandemic stage). To reduce inequalities and build resilience to epidemiological crises and consequent economic shocks, socioeconomic factors leveraging infection risk were identified from a comprehensive cross-section regression analysis covering 137 countries worldwide. Bridging some knowledge gaps concerning macro factors that mediate the transmission rates of infectious diseases, the empirical results of this study may inform on-going policy initiatives preventing the spread of viruses, including COVID-19. 

## 2. Factors Spreading Infectious Diseases

Assuming the heterogeneous spread of pandemics across countries because of diversified background contexts, propagation speed, dominance, and severity of COVID-19 justifies a deeper investigation on virus transmission patterns, clinical features, and risk factors for infection [10]. With the final objective of flattening the curve of infections at the required spatial scale, our study investigates the major socioeconomic factors at the base of within- and between-countries transmission of this novel infectious disease. 

With this perspective in mind, health authorities have designed protocols to safeguard public health in addition to the announcement of basic sanitation rules and the proliferation of specialized medical facilities to treat COVID-19 patients. These are the existence of adequate frontline healthcare personnel, the increase in the number of hospital beds and intensive care units, rising availability of diagnostic tests, and proliferation of medical supplies in designated hospitals, including personal protective equipment and ventilators. Based on the aforementioned analysis, the number of doctors is used here as a proxy of the existence of medical staff, in connection with other socioeconomic factors [11].

Countries’ discrepancies regarding activity of testing and tracking for COVID-19 were also considered here. More specifically, tests validate the number of officially reported coronavirus cases [8]. However, some countries only test people admitted to hospitals or based on restricted protocols; in other contexts, testing has intensified during the pandemic. Therefore, some of the former available data are an underestimation of the actual spread of COVID-19. Moreover, disadvantaged socioeconomic background is ubiquitously linked to susceptible immune system and mortality [12]. In particular, people with inferior socioeconomic status are demonstrated to be more vulnerable to infectious disease outbreaks [13]. Their social and economic context is either directly or indirectly associated with weak immune response and reduced life expectancy via several pathways [14]. Direct causes include poor nutrition and inability to access *healthcare services, which are often connected with* rising comorbidity [15]. *Implicit factors impacting (socioeconomically vulnerable) people due to the poor education they receive, have constrained the development of* health-related behaviours. Living in cramped neighbourhoods, overcrowded accommodation, and poor housing conditions may suppress the immune system, and persisting economic inequalities reduce the prospects of upward movement in the social hierarchy [16].

Consequently, socioeconomic deprivation seems to be inextricably linked with the COVID-19 pandemic. Disease spread will depend upon the specific local infrastructures and socioeconomic inequalities in each context. For example, densely populated neighbourhoods and vulnerable groups, such as refugee populations who reside in dedicated camps, are more susceptible to virus attacks due to the congested living conditions. Wealth and income level (via Gross Domestic Product per capita) was used in the study to assess the level of economic development in a country as more poor countries encounter barriers to basic sanitation facilities and decent hygienic conditions [17]. 

Additional variables shaping the spread of COVID-19 include population size and density. On the one hand, it was assumed that constrained population movements can significantly mitigate the prevalence of influenza [18]. On the other hand, trade growth may encourage disease transmission [19]. Higher income levels are indirectly associated with more intense trade and rising travelling population flows [5]. In addition, areas with high GDP per capita have more social interactions and increased domestic and international flight connections, as a result of expanded cross-country economic transactions and relationships. As a result, air travel passengers are considered a proxy of economic growth of interest when studying the spreading mechanisms of COVID-19 [20]. This transportation mode has largely contributed to the accelerated virus spread even across distant continents [21].

The role of population age structure in the pandemic spread has been extensively discussed in earlier literature [22]. Despite the fact that people of all ages can be infected by COVID-19, older people seem to be more vulnerable since they often have underlying medical problems. Therefore, age is an important feature that requires particular attention in the COVID-19 pandemic. The elderly (and more specifically the age group above 60 years) are over-represented among COVID-19 fatal outcomes [23]. Ageing populations may have weaker defensive health mechanisms to cope with the stress induced by the disease [24]. Gender is assumed to be another significant variable, since men are demonstrated to be severely affected by COVID-19, likely *more than* women [25].

Smoking is an additional risk factor of developing a severe form of COVID-19. Moreover, smoking is—at least indirectly—associated with the above-mentioned threat, as an increased prevalence of smoking is related with the occurrence of cardiovascular and respiratory diseases [26]. Smokers seem to be more susceptible towards severe forms of COVID-19 and appear to have a higher mortality rate [27]. In addition, climate conditions—and especially low air humidity and mild temperatures (e.g., dry–hot climate)—are assumed to be negatively associated with the spread of Coronaviruses [28], including COVID-19 [29,30].

## 3. Methodology

All the variables investigated in this study were derived from a collection of official statistics (and other well-known international data sources) for a sample of 137 countries worldwide. As far as time coverage is concerned, it must be pointed out that data from the most recent available year were employed. The *number* of *confirmed coronavirus cases per million people at a specific time point* (30 April 2020) was released by the Centre for Systems Science and Engineering (CSSE) at Johns Hopkins University. The same data source has provided the number of tests used to track coronavirus affected populations per million people by country. Sex ratio, population density (inhabitants per km^2^ of land area), and the share of population aged 65 as a percentage of total population, taken as a proxy of ageing, were derived from the World’s Bank database of development indicators. 

To assess within- and between-countries mobility and, as a result, the increased probability of virus transmission, the number of air transport passengers compiled by the World Bank and published in the above-mentioned database was used. To estimate the level of economic development in each country, log-transformed gross domestic product per capita (GDP) in purchasing power parity was used. This variable was derived from the World Economic Outlook (WEO) database of the International Monetary Fund (IMF). Data regarding the average annual temperature and air humidity were also considered. Total deaths and hospitalized patients were included in the analysis. In order to capture the level of technological development and research infrastructure in a country, research and development expenditure as a percentage of GDP was used. In addition, death rate per 1000 people was included in empirical estimates in addition to health expenditures, which was used as a proxy of health infrastructures, as well as the density of medical doctors per 1000 people as a proxy of health workforce. 

Data on cigarette consumption were derived from the World Health Organization. A morbidity indicator (an unweighted average of deaths caused in each country by serious illnesses and, more specifically, cardiovascular diseases, diabetes, chronic respiratory diseases, and cancer) was finally considered. In the case of patients with the abovementioned (pre-existing) medical conditions, the presence of COVID-19 drastically increases the likelihood of death [31]. 

An exploratory approach based on multiple stepwise linear regression models [32,33,34]) was run to explain the spatial variability separately in three dependent variables (i.e., the total number of detected coronavirus cases in each country, hospitalized patients, and people died with a COVID-19 diagnosis) with an extended set of predictors. Equation forms, results of the analysis, and technical (statistical) details are provided in the following section. Significant predictors were identified based on the results of a Fisher–Snedecor F statistic [35,36,37]). The probability level was fixed at 0.05 when testing statistical significance [38,39,40]). The SPSS (Statistical Package for Social Sciences) software (IBM, Chicago, IL, USA) was used when performing data analysis [41,42,43].

## 4. Results

### 4.1. Total Cases

A multiple stepwise (linear) regression model based on Equation (1):

Total cases = tests + male population + population density + population aged 65 and over + air transport passengers + ln(GDP) + average temperature + humidity(1)

demonstrated how three variables (tests per million, population density, and *air passengers*) significantly affect the dependent variable (Table 1). More specifically, Model 1 indicates that more than 50% of the variance of diagnosed coronavirus cases can be explained by the number of tests performed in each country. Air transport passengers contributed 6.3% in the equation’s goodness of fit. Model (3) reaches a very good total explanatory performance (adj-R^2^_TESTS, DENS, AIR_ = 0.72). Therefore, from Model 3, it emerges that tests per million, population density, and air passengers carried explain 74.1% of the variance of verified coronavirus infected patients. By using predictors in Equation (1), the following regression model was obtained (Model 3 of Table 1):

Total cases = 0.256 + 0.047 tests + 0.409 population density + 0.637 air transport passengers.

Among other factors, the unexplained part of the variance can be attributed to variables expressing social relationships that are not included in the analysis because of limited data availability within the global sample. More specifically, these are country-specific factors dealing with local culture and attitudes associated with frequency of human interactions, indirectly related to the virus’ spread. In addition, traditional habits and close family ties especially with older members may affect the transmission speed in a given community. An additional aspect of the issue is that governments differ in the timing of implementing social distancing and lockdown measures, which affects their overall efficacy and impact. Moreover, between the enforcement of quarantine policies, changing social response efforts, and identified COVID-19 cases, there was a latency period of largely heterogeneous length. As a result, not only the application of stringent government measures but also the restrictions’ timing and length were crucial regarding the spread of COVID-19. It should be pointed out that the effect of economic development expressed with income may be easily delineated using air travel intensity, because of the high intrinsic correlation between these two indicators.

Whereas the model summary table examines the predictors taken as a set, the model’s coefficients delineate the individual impact of each predictor (Table 2). According to the related regression coefficients (respectively 0.047, 0.409, 0.637), the number of tests, population density, and air transport passengers indicate that, in a given country, the higher the number of tests per million population is, the higher population density exists, and the more air transport passengers are carried, the higher is the number of total cases and the lower is the number of undetected infections (Table 2). 

Regression diagnostics, such as tolerance statistics (very high) and a very low variance inflation factor (VIF < 5) for all independent variables, indicate the absence of serious multicollinearity problems. The conditional index for the last dimension is slightly lower than 15 and the eigenvalue, not exactly equal to 0, indicates negligible multicollinearity. Variables such as tests and air passengers are finally associated with high variance proportions in the last dimension. The Durbin–Watson test did not indicate autocorrelation, as d = 1.822 > d_U_ = 1.74 and 4-d = 2.178 > d_U_ = 1.74 with explanatory variables K = 3, a = 0.05, and *n* = 137 (Table 3). Additionally, studentized deleted residuals seem to follow the normal distribution according to all statistics and tests (skewness statistic = 0.091, std. error = 0.185, kurtosis statistic = 1.125, std. error = 0.367), documenting the appropriateness of the statistical technique adopted in our study.

### 4.2. Total Deaths

Similarly with the previous analysis, factors affecting the total number of deaths that occurred due to coronavirus were modelled in each country according to the following form:

Total deaths = tests + male population + research and development expenditure (% GDP) + population aged 65 and over + ln(GDP) + death rate + physicians + health expenditure (% GDP) + cigarette consumption + morbidity(2)

Empirical results indicate that three variables significantly affect the dependent variable (Table 4): tests per million people, population aged 65 years and over, male population. The number of tests explains most of the variation of the dependent variable (75.3%). While being a factor related to increased coronavirus fatal outcomes, the importance of sex ratio as predictor can be indirectly explained by the larger cigarette consumption by males in respect to that of females [44].

According to their regression coefficients (−0.118, 0.209, 0.703), tests per million population, population aged 65 years and over (per cent share in total population), and sex ratio suggest that, in a given country, the lower the number of tests per million population and the more aged the population, the higher the number of deaths per million population and vice versa (Table 5).

Tolerance statistics are high and VIFs are low (VIF < 10) for all independent variables, outlining negligible multicollinearity (Table 6). Conditional index for the last dimension is low (5.590 < 15) and eigenvalue is near (but not equal to) 0, both indicating absence of serious multicollinearity issues. Population age is the only variable associated with high variance proportions in the last dimension. The Durbin–Watson test did not indicate autocorrelation, as d = 2.008 > d_U_ = 1.72 and 4-d = 1.992 > d_U_ = 1.72 with explanatory variables K = 3, a = 0.05, and *n* = 137. Additionally, studentized deleted residuals follow the normal distribution in accordance with all statistics and tests (skewness = 0.015, std. error = 0.185, kurtosis = 1.90, std. error = 0.367). The aforementioned results suggest that the model has an excellent total explanatory performance, as the coefficient of determination R^2^_TESTS, AGE, MALE_ = 95.9%, the coefficients appear to have socioeconomic significance and the assumptions for the model acceptance are satisfied. 

### 4.3. Hospitalized Patients

According to the previous analysis, the equation illustrating factors that affect the total number of hospitalized coronavirus patients in each country has the following form:

Hospitalized patients = tests + male population + research and development expenditure (% GDP) + population aged 65 and over + ln (GDP) + death rate + physicians + health expenditure (% GDP) + cigarette consumption + morbidity(3)

Three variables were found to affect the dependent variable in a significant way (Table 7): tests per million people, the number of employed medical doctors, and *morbidity*. The number of tests used explains a significant part of the variability of the dependent variable (50%). The effect of age can be associated with morbidity, because of high pair-wise correlation (Table 8). Older people suffer more often from *life-threatening diseases* [45]. Tolerance statistics are high and VIFs are low (VIF < 10) for all independent variables, indicating negligible multicollinearity (Table 9). Conditional index for the last dimension is 18.484, slightly higher than 15, and the eigenvalue is near 0, both indicating nonserious multicollinearity. The tests variable is the only one associated with high variance proportions in the last dimension. The Durbin–Watson test did not indicate autocorrelation, as d = 2.059 > d_U_ = 1.74 and 4-d = 1.941 > d_U_ = 1.74 with explanatory variables K = 3, a = 0.05, and *n* = 137. Additionally, studentized deleted residuals variable seems to face some but not very serious kurtosis problems (skewness statistic = −0.068, std. error = 0.185, kurtosis statistic = 1.152, std. error = 0.367). The aforementioned results suggest that the model has a good explanatory performance, as the coefficient of determination R^2^_TESTS, PHYSICIANS, MORBIDITY_ = 0.819. The coefficients appear to have statistical and conceptual significance and the assumptions for the model acceptance are satisfied.

## 5. Discussion

As the global economy continues to absorb the shock of the coronavirus pandemic, research is required to overcome daunting challenges and assess the transmission dynamics of the virus, considering socioeconomic variables as significant factors of change [6,7,31]. The empirical results of our study have implications for active global efforts in the containment of COVID-19. They could also complement epidemiologic studies towards the direction of curbing the spread of infectious diseases in the future, for both COVID-19 and other similar pandemics. Socioeconomic conditions are ubiquitous factors affecting life expectancy and mortality [2,24,46]. Moreover, the availability of healthcare resources and their coordination are critical parts in the containment of infectious diseases [47].

Assuming interpretative models exclusively grounded on epidemiological predictors as providing a partial view on the latent mechanisms of COVID-19 spread, socioeconomic factors contribute to delineate the extent of the infection in the community, in addition with the epidemiological predictors. These results suggest how, in an effort to break the chain of infections, the social nature of prevention and control measures required the active enrolment of local communities [3,14,15,48]. Hence, understanding the association between the pattern of spread of the epidemic and responses to COVID-19 is particularly important in all countries and especially those marked by extensive socioeconomic disparities [49].

Regression results indicate that increased diagnostic capacity via COVID-19 testing is associated with the (more or less evident) containment of virus’ spreading and registered deaths. The same applies for the improvement of living conditions as reflected in the level of population density. The existence of medical personnel is associated with the *retrieval* of *patients* with *COVID-19*. The significance of smoking is probably associated with the effect of morbidity, as smokers are more likely to have chronic health conditions, and the pair-wise correlation between these variables was found to be rather intense. The effect of the environmental factors is still blurry [9]. Moreover, the results of the current study are in line with studies supporting the view that stringent quarantine, massive lockdown, and other public health measures imposed by governments worldwide significantly impeded the transmission rate of COVID-19 [50].

As a response to the global shock of COVID-19, social protection and medical assistance programmes should be scaled up and their coverage extended significantly. Moreover, many countries are urged to upscale their health infrastructure, improving equipment, resources, and skills needed to fight the spread of coronaviruses, in turn protecting the livelihoods of their people [8]. Furthermore, in order to strengthen public health preparedness and deal with future public health risks, equitable public health prevention measures should be developed. More specifically, all-embracing access to healthcare services should be strengthened along with the development of sustainable health systems [10]. Therefore, socioeconomic factors should be taken into consideration when implementing public health interventions [51].

As COVID-19 spreads worldwide, it is essential to employ data on socioeconomic determinants for a more targeted approach aimed at identifying high-risk populations. A refined comprehension of the role of socioeconomic attributes (apart from the clinical characteristics of COVID-19) sheds further light into future prevention measures against similar infectious diseases [52]. With this perspective in mind, conceptual and technical limits of this kind of study should be definitely clarified and possibly solved, including the lack of data for social relations/networks, in turn providing a comprehensive background that compares different policy strategies applied at the local scale (e.g., lockdown time scheduling) and temporal lags in pandemic spread—allowing countries affected later by the virus to learn more rapidly and effectively from the mistakes of countries affected earlier [4].

In such research directions, the importance of social, cultural, demographic, and economic capitals forming an individual’s health capital and the implications for COVID-19 pandemic management and control should be better clarified, considering earlier studies, e.g., from the perspective of Bourdieu’s theory of capitals [53]. Seemingly contradictory health behaviours such as smoking—and their intrinsic implications for coronaviruses’ spread—can be also explained in light of this theory [54].

## 6. Conclusions

In order to develop appropriate public health prevention measures, effective guidelines and interventions and data sources with comprehensive socioeconomic measures are particularly important. As a result, to increase policy effectiveness, socioeconomic background contexts should be extensively investigated in order to advance our common knowledge in the field.

## Figures and Tables

**Table 1:** Model summary (total infections as dependent variable).

Model	R	R Square	Adjusted R Square	Std. Error of the Estimate	Change Statistics					Durbin–Watson
					R Square Change	F Change	df1	df2	Sig. F Change	
1	0.716 ^a^	0.513	0.500	832.8039	0.513	38.945	1	37	0.000	
2	0.823 ^b^	0.677	0.659	687.1179	0.165	18.353	1	36	0.000	
3	0.861 ^c^	0.741	0.718	624.9052	0.063	8.525	1	35	0.006	1.822

^a^ Predictors: (constant), tests per million; ^b^ predictors: (constant), tests per million, population density (people per sq. km of land area); ^c^ predictors: (constant), tests per million, pop. density (people per km^2^ of land area), air transport passengers;

**Table 2:** Model coefficients.

Model		Unstandardized Coefficients		Standardized Coefficients	t	Sig.	Correlations			Collinearity Statistics	
		B	Std. Error	Beta			Zero order	Partial	Part	Tolerance	VIF
1	(Constant)	41.127	94.948		1.728	0.032					
	Tests per million	0.046	0.007	0.716	6.241	0.000	0.716	0.716	0.716	1.000	1.000
2	(Constant)	40.751	89.672		1.762	0.035					
	Tests per million	0.040	0.006	0.615	6.304	0.000	0.716	0.724	0.597	0.942	1.062
	Population density	0.465	0.109	0.418	4.284	0.000	0.567	0.581	0.406	0.942	1.062
3	(Constant)	0.256	0.601		2.651	0.000					
	Tests per million	0.047	0.006	0.732	7.519	0.000	0.716	0.786	0.647	0.782	1.279
	Population density	0.409	0.101	0.357	4.062	0.000	0.567	0.566	0.350	0.907	1.103
	Air transport passengers	0.637	0.741	0.277	2.920	0.006	0.029	0.443	0.251	0.825	1.212

Dependent variable: total cases per million population.

**Table 3:** Collinearity diagnostics of the final stepwise model.

Model	Dimension	Eigenvalue	Condition Index	Variance Proportions			
				(Constant)	Tests	Population Density	Air Passengers Carried
3	1	3.185	1.000	0.01	0.00	0.02	0.00
	2	0.701	2.131	0.00	0.00	0.45	0.01
	3	0.099	5.672	0.86	0.01	0.49	0.05
	4	0.014	14.882	0.12	0.98	0.03	0.94

Dependent variable: total cases per million population.

**Table 4:** Model summary (total deaths as dependent variables).

Model	R	R Square	Adjusted R Square	Std. Error of the Estimate	Change Statistics					Durbin–Watson
					R Square Change	F Change	df1	df2	Sig. F Change	
1	0.882 ^a^	0.778	0.753	49.0011	0.778	31.451	1	9	0.000	
2	0.937 ^b^	0.878	0.847	38.5258	0.100	6.560	1	8	0.034	
3	0.970 ^c^	0.941	0.916	28.5879	0.063	7.529	1	7	0.029	2.008

^a^ Predictors: (constant), tests; ^b^ predictors: (constant), tests, population aged 65 and over (% of total population); ^c^ predictors: (constant), tests, population aged 65 + (% total population), population, male (% total population).

**Table 5:** Model coefficients.

Model		Unstandardized Coefficients		Standardized Coefficients	t	Sig.	Correlations			Collinearity Statistics	
		B	Std. Error	Beta			Zero Order	Partial	Part	Tolerance	VIF
1	(Constant)	1.333	19.192		2.032	0.003					
	Tests	−0.011	0.002	−0.882	−5.608	0.000	−0.885	−0.842	−0.761	1.000	1.000
2	(Constant)	12.861	16.075		0.800	0.447					
	Tests	−0.013	0.002	−0.804	−7.577	0.000	−0.882	−0.937	−0.943	0.870	1.149
	Population aged 65+	−0.091	0.035	0.339	2.561	0.034	0.022	0.671	0.317	0.868	1.153
3	(Constant)	0.849	0.091		2.835	0.025					
	Tests	−0.118	0.022	−0.827	−7.805	0.000	−0.882	−0.947	−0.716	0.752	1.372
	Population aged 65+	0.209	0.050	0.783	4.138	0.004	0.022	0.843	0.380	0.835	1.250
	Population, male	0.703	0.074	0.551	2.744	0.029	0.544	0.720	0.252	0.909	1.391

Dependent variable: total deaths per million population.

**Table 6:** Collinearity diagnostics of the final stepwise model.

Model	Dimension	Eigenvalue	Condition Index	Variance Proportions			
				Constant	Tests	Population Aged	Population Male
3	1	2.356	1.000	0.02	0.05	0.03	0.00
	2	0.568	2.036	0.00	0.50	0.11	0.00
	3	0.109	3.018	0.03	0.40	0.24	0.04
	4	0.075	5.590	0.17	0.45	0.86	0.27

Dependent Variable: deaths per million population.

**Table 7:** Model summary (hospitalized patients as dependent variable).

Model	R	R Square	Adjusted R Square	Std. Error of the Estimate	Change Statistics					Durbin–Watson
					R Square Change	F Change	df1	df2	Sig. F Change	
1	0.725 ^a^	0.526	0.478	0.069029	0.526	11.087	1	10	0.008	
2	0.868 ^b^	0.753	0.698	0.052521	0.227	8.274	1	9	0.018	
3	0.932 ^c^	0.868	0.819	0.040713	0.115	6.978	1	8	0.030	2.059

^a^ Predictors: (constant), tests per million; ^b^ predictors: (constant), tests per million, physicians; ^c^ predictors: (constant), tests per million, physicians, morbidity.

**Table 8:** Model coefficients.

Model		Unstandardized Coefficients		Standardized Coefficients	t	Sig.	Correlations			Collinearity Statistics	
		B	Std. Error	Beta			Zero Order	Partial	Part	Tolerance	VIF
1	(Constant)	0.049	0.026		1.905	0.086					
	Tests per million	0.820	0.102	0.904	8.034	0.000	0.828	−0.943	0.892	0.974	1.027
2	(Constant)	0.056	0.018		1.989	0.095					
	Tests per million)	0.750	0.170	0.828	4.424	0.002	0.828	0.818	0.709	1.000	1.000
	Physicians	0.085	0.020	0.471	4.188	0.003	0.324	0.829	0.465	0.974	1.027
3	(Constant)	0.037	0.027		1.381	0.205					
	Tests per million	0.879	0.048	0.838	18.302	0.000	0.828	0.990	0.933	0.925	1.081
	Physicians	0.067	0.010	0.555	6.993	0.000	0.324	0.935	0.357	0.883	1.132
	Morbidity	−0.209	0.050	−0.350	−4.138	0.004	0.022	−0.843	−0.380	0.235	1.250

Dependent variable: recovered patients.

**Table 9:** Collinearity diagnostics of the final stepwise model.

Model	Dimension	Eigenvalue	Condition Index	Variance Proportions			
				Constant	Tests	Physicians	Morbidity
3	1	3.306	1.000	0.00	0.00	0.01	0.01
	2	0.575	2.397	0.00	0.00	0.10	0.21
	3	0.109	5.503	0.03	0.04	0.54	0.40
	4	0.010	18.484	0.97	0.96	0.35	0.38

Dependent variable: recovered patients.

## Data Availability

https://coronavirus.jhu.edu/map.html (accessed on 7 June 2021).
